# Prevalent Synergy and Antagonism Among Antibiotics and Biocides in *Pseudomonas aeruginosa*

**DOI:** 10.3389/fmicb.2020.615618

**Published:** 2021-02-04

**Authors:** Franziska Pietsch, Gabriele Heidrich, Niclas Nordholt, Frank Schreiber

**Affiliations:** Division of Biodeterioration and Reference Organisms (4.1), Department of Materials and the Environment, Federal Institute for Materials Research and Testing (BAM), Berlin, Germany

**Keywords:** synergy, antagonism, suppression, biocides, antibiotics, pseudomonas aeruginosa, CTAB (cetyltrimethylammonium bromide), Povidone-iodine (PVP-I)

## Abstract

Antimicrobials can exert specific physiological effects when used in combination that are different from those when applied alone. While combination effects have been extensively mapped for antibiotic-antibiotic combinations, the combination effects of antibiotics with antimicrobials used as biocides or antiseptics have not been systematically investigated. Here, we investigated the effects of combinations of antibiotics (meropenem, gentamicin, and ciprofloxacin) and substances used as biocides or antiseptics [octenidine, benzalkonium chloride, cetrimonium bromide, chlorhexidine, Povidone-iodine, silver nitrate (AgNO_3_), and Ag-nanoparticles] on the planktonic growth rate of *Pseudomonas aeruginosa*. Combination effects were investigated in growth experiments in microtiter plates at different concentrations and the Bliss interaction scores were calculated. Among the 21 screened combinations, we find prevalent combination effects with synergy occurring six times and antagonism occurring 10 times. The effects are specific to the antibiotic-biocide combination with meropenem showing a tendency for antagonism with biocides (6 of 7), while gentamicin has a tendency for synergy (5 of 7). In conclusion, antibiotics and biocides or antiseptics exert physiological combination effects on the pathogen *P. aeruginosa*. These effects have consequences for the efficacy of both types of substances and potentially for the selection of antimicrobial resistant strains in clinical applications with combined exposure (e.g., wound care and coated biomaterials).

## Introduction

Combinatorial exposure to multiple antibiotics has been suggested to improve treatment outcome, especially for infections with multi-drug resistant bacteria (Tamma et al., 2012; Tyers and Wright, 2019). However, depending on the conditions, combination treatment may accelerate or delay resistance selection (Hegreness et al., 2008; Michel et al., 2008; Baym et al., 2015; Barbosa et al., 2018). Therefore, choosing the best combination requires knowledge on possible physiological interactions between the effects of the combined active substances; i.e., are their individual effects simply added, potentiated (synergy), or buffered (antagonism and supression) when used in combination (Baym et al., 2015). Combinatorial effects among antibiotics (Yeh et al., 2006; Brochado et al., 2018), antimicrobial peptides (Yu et al., 2016), and antibiotics with virulence factors (Rezzoagli et al., 2020) have been mapped extensively. In contrast, combinatorial effects of compounds used as disinfectants, preservatives, antiseptics, and as antimicrobial surface coatings (for simplicity from now on called biocides) with antibiotics received less attention (Brochado et al., 2018). However, there are several situations in which antibiotics and biocides can exert combination effects on microbes. These situations are related to clinical treatment schemes in which topical treatment of microbial infections with antiseptics is supported by systemic antibiotic dosing; for example, treatment of chronic wounds (Sibbald et al., 2007; Leaper et al., 2012) and implantation of medical devices coated with antimicrobials (von Eiff et al., 2005; Busscher et al., 2012; Moriarty et al., 2013). In addition, microbes might be exposed to combinations of antibiotics and biocides in certain environmental compartments such as hospital wastewater or wastewater treatment plants.

Here, we investigate combinatorial effects of antibiotics and biocides on the pathogen *Pseudomonas aeruginosa*. We chose *P. aeruginosa* because it is a prevalent pathogen in clinical situations in which antibiotics and antiseptics or biocides meet; i.e., it often occurs in wound infections (Percival et al., 2015; Serra et al., 2015), it is known to colonize medical implants (Percival et al., 2015), and it is known to be widespread in environmental compartments. We chose three antibiotics that are used to treat *P. aeruginosa* infections and seven biocides, all covering a range of classes, modes of action and applications (Table 1).

**Table 1:** Characteristics of antibiotics and biocides screened for interaction effects.

**Antibiotic**	**Class**	**Mode of action**	**Application**	^*^MIC	^*^^,^^#^**EC**_**50**_
Meropenem	Carbapenem; beta-lactam	Inhibition of bacterial cell wall synthesis	Broad spectrum; treatment of infections with MDR bacteria	0.075	0.061 (0.004)
Ciprofloxacin	Fluoroquinolone	Inhibition of DNA transcription and replication	Broad spectrum	1.5	0.22 (0.39)
Gentamicin	Aminoglycoside	Inhibition of protein synthesis	Broad spectrum; topical applications	0.5	0.30 (0.07)
**Biocide**					
Octenidine	Cationic surfactant	Binds to cell envelope; Interaction with enzymes and polysaccharides in the cell envelope; induces leakage in the cytoplasmic membrane	Antiseptic for skin disinfection and wound treatment	1	0.8 (0.3)
Benzalkonium chloride (BAC)	Cationic quaternary ammonium compound	Interacts with cell membranes, leading to disruption of membrane integrity and leakage of cellular content	Antiseptic ointments, drops, creams and sprays; antimicrobial coatings	25	21.4 (0.8)
Cetrimonium bromide (CTAB)	Cationic quaternary ammonium compound	Binding to lipid components of the cell membrane, causing membrane rupture and cell lysis	Antimicrobial coating and topical antiseptic in hygiene and pharmaceuticals	16	13.3 (0.8)
Chlorhexidine	Cationic biguanide	Binds to negatively charged bacterial walls, causing membrane disruption and leakage	Topical skin disinfectants, wound and burn dressings, coating of catheters	7.6	5.6 (0.2)
Povidone-iodine (PVP-I)	Halogen	Rapidly penetrates microorganisms, damaging proteins, nucleotides and fatty acids	Disinfectant in wound dressings	150	138 (5)
Silver nitrate (AgNO_3_)	Transition metal	Ability to bind FeS-clusters or thiol-groups of enzymes, inhibiting cellular functions such as electron transport chain; ROS formation	Topical antiseptic on moist skin, mucous and skin wounds	0.01	0.0058 (0.0006)
Silver nanoparticles (AgNP)	Transition metal	Similar to AgNO_3_	Antimicrobial coating on medical devices	0.03	0.026 (6.8)

* MIC and EC_50_ measured in M9 medium, concentration in μg ml^−1^.

# The brackets depict the standard error of the simultaneous dose-response fit of three biological replicates scaled with the square route of the reduced *χ*^2^.

## Materials and Methods

### Strains and Growth Conditions

The experiments were performed with *P. aeruginosa* MPAO1 obtained from Colin Manoil (Jacobs et al., 2003; Varadarajan et al., 2020). The ancestor strain was isolated from a wound in Melbourne, Australia, in 1954 (Holloway, 1955; Chandler et al., 2019). All assays were conducted in 200 μl volume in a polypropylene (PP; PP has lower binding for positively charged biocides as compared to polystyrene) 96-well microtiter plate incubated at 37°C with shaking (fast, orbital mode) in a plate reader (EPOCH2, Biotek) with readings for OD_600_ obtained in a 5 min interval. The inoculum was 10^5^ cells per ml and consisted of a pre-culture of exponential cells in M9 [5 × M9 minimal salts base from Formedium (final concentrations: 6.78 g L^−1^ Na_2_HPO_4_; 3 g L^−1^ KH_2_PO_4_; 0.5 g L^−1^ NaCl; 1 g L^−1^ NH_4_Cl) supplemented with 2 mM MgSO_4_, 100 μM CaCl_2_, 20 mM glucose, 25 μM FeCl_3_⋅6H_2_O, 4.95 μM ZnCl_2_, 2.1 μM CoCl_2_⋅6H_2_O, 2 μM Na_2_MoO_4_⋅2H_2_O, 1.7 μM CaCl_2_⋅2H_2_O, 2.5 μM CuCl_2_⋅2H_2_O, 2 μM H_3_BO_3_] that were stored at −80°C until use (each freezer stock was only thawed once). Defined M9 medium was chosen for the experiments instead of nutritionally-rich media such as Mueller-Hinton broth (recommended for standard antimicrobial susceptibly testing) to generate data that can be reproduced despite apparent differences between different Mueller-Hinton brands (Ahman et al., 2020), to minimize interactions between medium and biocides, and to allow tracing of the molecular mechanism relative to general metabolism in a balanced medium with a single carbon and nitrogen source.

### Determination of MIC and EC_50_

Minimum inhibitory concentrations (MIC) were determined by broth microdilution according to the standard method described previously (Wiegand et al., 2008) with the following modifications: M9 was used as growth medium, microtiter plates were incubated under shaking conditions, and pre-cultures were used from exponential cultures that were stored at −80°C and thawed on ice prior to the experiment. Wells were inoculated with 5 × 10^3^ CFU per well containing 200 μl medium. OD_600_ values were recorded in a microplate reader (BioTek EPOCH 2, intermediate linear shaking, 37°C) every 5 min for 24 h. All measurements were run at least in triplicate. The lowest concentration for which no growth was observed in all replicates based on OD_600_ after 24 h was set as MIC. Wells containing only M9 and the specific antimicrobial substance without cells served as background control. In parallel, half-maximal effective concentrations (EC_50_) were derived from maximum growth rates at different antimicrobial concentrations. The growth rate was calculated from the increase in OD_600_ over time by inferring the time derivative using Gaussian processes implemented as published code (Swain et al., 2016) in Python 3.6 and fitting a dose-response model to the data of the combined three biological replicates (DoseResp fit, Origin 2019 v9.6, OriginLab).

### Combination Effects

**Figure 1:**
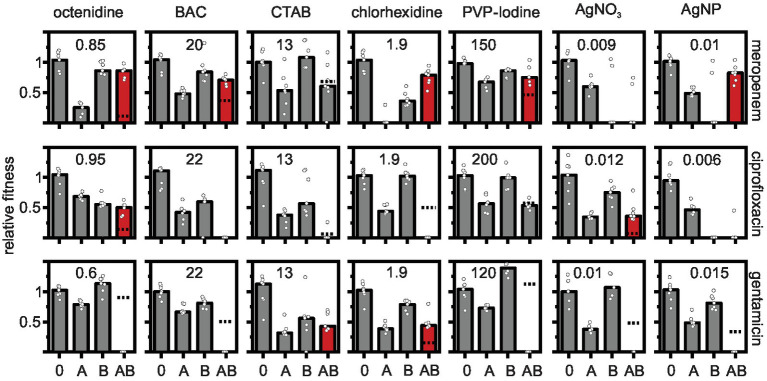
Effects of antibiotic-biocide combinations on the relative fitness of *Pseudomonas aeruginosa* MPAO1. Relative fitness was measured as the growth rate in the absence of antimicrobial (0), in the presence of antibiotic (A), in the presence of biocide (B), and in the presence of a combination of antibiotic with biocide (AB) normalized by the growth rate in the absence of antimicrobial. Concentrations of antibiotics were 0.06 μg ml^−1^ for meropenem; 0.6 μg ml^−1^ for ciprofloxacin; and 0.18 μg ml^−1^ for gentamicin. The concentrations of the biocides (in μg ml^−1^) are depicted in each panel. The dashed lines depict the combined effect of both antibiotic and biocide predicted under the Bliss independence model. The predicted fitness under combinatorial treatment is zero in panels in which the dashed line is absent. Combinations that show a higher measured fitness in the presence of the combination as compared to the predicted fitness are typically antagonistic (shown as red bars). Combinations that show a lower measured fitness in the presence of the combination as compared to the predicted fitness are synergistic (dashed line displayed, but measured fitness in combination typically equals zero). Additive combinations are shown as gray bars. Significance was tested with a one-sample *t*-test on the Bliss interaction score as described in the main text (see Figure 2). The bars represent the median and the white circles represent individual measurements of seven biological replicates (in some treatments one or two replicates had to be omitted due to difficulties in fitting the growth curves).

A three-step procedure was used to identify physiological interactions of antibiotics and biocides. First, we determined the minimal inhibitory concentrations MICs and EC_50_ of all compounds in isolation (see section above, Table 1). Secondly, for each antibiotic-biocide combination, we determined the growth rates of seven biological replicate cell populations in M9 minimal medium (i) without antimicrobials, (ii) with the antibiotic alone at approximately the EC_50_, (iii) with five concentrations close to the EC_50_ of the biocide alone (see Supplementary Table S1), and (iv) with five combinations of the antibiotic at approximately EC_50_ supplied simultaneously with the biocide at five concentrations close to the EC_50_ (exactly those as measured in the “biocide alone” treatment). Each antibiotic-biocide combination was tested in parallel on the same day to minimize day-to-day variations in growth rate effects of antibiotics, which emerged due to steep dose-response curves and which were especially apparent for meropenem (Figure 1). Thirdly, we calculated the interaction effects of the combinations based on the Bliss independence model (Bliss, 1939).

### Calculations and Statistics

The Bliss independence model was chosen because it is recommended for compounds that affect different target sites (Baeder et al., 2016) as expected for antibiotics and biocides. An alternative model is Loewe additivity including the popular checkerboard assay with calculation of the factorial inhibition concentration index (Loewe, 1953; Hallander et al., 1982). It has been shown that Bliss independence (response additivity) and Loewe (dosage additivity) give robust results when calculating effects of pairwise combinations of a large array of antibiotics (Russ and Kishony, 2018). The Bliss interaction score (S) was defined as the difference between the measured combined effect (E_AB_) and the summed, single effects of the antibiotics (E_A_) and the biocides (E_B_); S = E_AB_−(E_A_ + E_B_) (Russ and Kishony, 2018). An additive definition of the Bliss model was used because the effects were based on growth rates rather than yield measurements (Baeder et al., 2016; Russ and Kishony, 2018). All effects of antimicrobials or their combinations (E_i_) were calculated as the difference between the measured growth rate without antimicrobial (g_0_) and the growth rates with antimicrobials (g_i_), relative to the growth rate without antimicrobial; E_i_ = (g_0_−g_i_)/g_0_ (Russ and Kishony, 2018). Bliss interactions scores of each antibiotic-biocide combination were calculated for each of the seven biological replicates separately and tested for normality with a Kolmogorov-Smirnov test (normality was not rejected for any biocide-antibiotic combination at *p* < 0.05, Origin 2019 v9.6). Next, the Bliss interactions scores of each antibiotic-biocide combination were tested for a significant deviation from zero (additive combination effect) with a one-sample *t*-test (Origin 2019 v9.6, OriginLab, at p < 0.05).

## Results and Discussion

**Figure 2:**
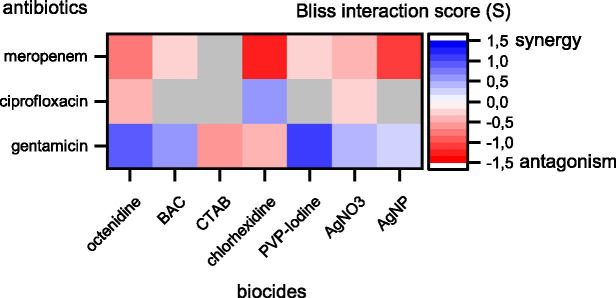
Synergy and antagonism between antibiotics and biocides based on Bliss interaction scores. Red colors depict antagonistic and blue colors depict synergistic interactions. Combinations displayed in colors were significant according to a one-sample *t*-test (*p* < 0.05). Combinations displayed in gray were not significant. Note that our assay cannot fully exclude synergy or antagonism for those combinations shown in gray. These interactions might be detectable if more combinations of different concentrations are tested.

The results show prevalent combination effects when antibiotics and biocides were applied simultaneously with synergy occurring six times and antagonism occurring 10 times among the 21 screened combinations (Figures 1, 2). The effects are specific to the antibiotic-biocide combination, with some apparent patterns. Interactions of biocides with meropenem were predominantly antagonistic (6 of 7, except for cetrimonium bromide (CTAB), which showed no significant interaction), while interactions of biocides with gentamicin were predominately synergistic (5 of 7, except for CTAB and chlorhexidine, which both were antagonistic). For ciprofloxacin, we detected three significant interactions; octenidine, silver nitrate (AgNO_3_; antagonism), and chlorhexidine (synergy).

Biocides with similar chemistries mostly showed consistent interactions with different antibiotics. For example, cationic surfactants (octenidine) and cationic quaternary ammonium compounds [QACs, here benzalkonium chloride (BAC)] were antagonistic with meropenem and synergistic with gentamicin. However, CTAB, which is also a cationic QAC, did not show an interaction with meropenem and was antagonistic with gentamicin. Moreover, AgNO_3_ and silver nanoparticles (AgNP) were antagonistic with meropenem and synergistic with gentamicin. The observed interactions between antibiotics and silver are consistent with previous reports that showed synergistic effects between aminoglycosides and silver on killing of planktonic *Escherichia coli* (Morones-Ramirez et al., 2013) and biofilms of *P. aeruginosa* (Habash et al., 2017), and that showed no effect on planktonic growth of *P. aeruginosa* ATCC 10145 for the ciprofloxacin-AgNP combination (Markowska et al., 2014). In contrast, the antagonistic effect between AgNP and meropenem apparent in our data has not been observed previously in *P. aeruginosa* ATCC 10145 (Markowska et al., 2014). In addition, a strain-specific, synergistic effect between chlorhexidine and gentamicin for clinical isolates of *P. aeruginosa* has been described previously (Barnham and Kerby, 1980), while our data show antagonistic effects on strain MPAO1.

Here, we present the first comprehensive screen of physiological interactions of antibiotic-biocide combinations in *P. aeruginosa*. This data will provide the basis for designing improved treatment protocols in which biocides/antiseptics and antibiotics are used in combination. This might be particularly important in situations in which these antimicrobials establish concentration gradients. Such gradients may lead to the establishment of relevant combinatorial concentrations that then might lead to combination effects on growth. The results provide the basis for future work that should focus on (i) confirming the combination effects with other established concepts such as Loewe additivity (including the factorial inhibition concentration; Hallander et al., 1982), (ii) expand the screen for combination effects to other antibiotics (e.g., antimicrobial peptides) and antiseptics/biocides, (iii) mapping the occurrence of the combination effects across *P. aeruginosa* clinical isolates and other pathogens, and (iv) investigating the molecular mechanism behind the combination effects by gene expression or knockout studies. Moreover, future research should explore potential evolutionary consequences of the physiological interaction effects between antibiotics and biocides. This is relevant because the nature of the physiological interaction (synergy or antagonism) has been shown to underpin selection for or against antimicrobial resistant strains in competition with sensitive strains exposed to combinations of antimicrobials (Chait et al., 2007, 2016; Baym et al., 2015; Rezzoagli et al., 2020).

## Data Availability Statement

The raw data supporting the conclusions of this article will be made available by the authors, without undue reservation.

## Author Contributions

FS conceived the study. FP and FS designed the experiments. FP and GH performed the experiments. FP, GH, NN, and FS analyzed the data. FS wrote the manuscript with input from all co-authors. All authors contributed to the article and approved the submitted version.

### Conflict of Interest

The authors declare that the research was conducted in the absence of any commercial or financial relationships that could be construed as a potential conflict of interest.
