# Supplementary material for: Prevalent Synergy and Antagonism Among Antibiotics and Biocides in Pseudomonas aeruginosa
Source: Front Microbiol. 2021 Feb 4;11:615618. doi: 10.3389/fmicb.2020.615618 (PMC7889964; doi:10.3389/fmicb.2020.615618)
Supplement: Supplementary file 1 [file Data_Sheet_1.pdf]

Supplementary Information

Prevalent synergy and antagonism among antibiotics and biocides in *Pseudomonas aeruginosa*

Franziska Pietsch[a], Gabriele Heidrich[a], Niclas Nordholt[a] and Frank Schreiber[a#]

[a] Division of Biodeterioration and Reference Organisms (4.1), Department of Materials and Environment, Federal Institute for Materials Research and Testing (BAM), Berlin, Germany

Table S1. Biocide concentrations used in combination assays.

Biocide	Biocide concentration (µg mL^{-1}) used in assay with:		
	Meropenem	Ciprofloxacin	Gentamicin
Octenidine	0.65	0.65	0.6
	0.75	0.75	0.75
	0.85	0.85	0.9
	0.9	0.9	1.05
	0.95	0.95	1.2
Benzalkonium chloride (BAC)	18	21	18
	20	22	20
	22	23	22
	24	24	24
	26	25	26
Cetrimonium bromide (CTAB)	11	13	13
	13	14	14
	15	15	15
	17	16	16
	19	17	17
Chlorhexidine	1.9	1.9	1.9
	3.8	3.8	3.8
	5.7	5.7	5.7
	7.5	7.5	7.5
	9.4	9.4	9.4
Povidone-iodine (PVP-I)	130	150	120
	135	200	140
	140	250	160
	145	300	180
	150	350	200
Silver nitrate ($AgNO_3$)	0.007	0.007	0.01
	0.009	0.009	0.03
	0.01	0.01	0.05
	0.012	0.012	0.07
	0.014	0.014	0.09
Silver nanoparticles (AgNP)	0.0025	0.002	0.01
	0.005	0.004	0.015
	0.0075	0.006	0.02
	0.01	0.008	0.025
	0.015	0.01	0.03
